# LIM kinase 1 - dependent cofilin 1 pathway and actin dynamics mediate nuclear retinoid receptor function in T lymphocytes

**DOI:** 10.1186/1471-2199-12-41

**Published:** 2011-09-16

**Authors:** Mohammad Ishaq, Bor-Ruei Lin, Marjorie Bosche, Xin Zheng, Jun Yang, Dawei Huang, Richard A Lempicki, Ven Natarajan

**Affiliations:** 1 Laboratory of Molecular Cell Biology, SAIC-Frederick, National Cancer Institute, Frederick, MD 21702, USA; 2 Laboratory of Immunopathogenesis and Bioinformatics, SAIC-Frederick, National Cancer Institute, Frederick, MD 21702, USA

## Abstract

**Background:**

It is known that retinoid receptor function is attenuated during T cell activation, a phenomenon that involves actin remodeling, suggesting that actin modification may play a role in such inhibition. Here we have investigated the role of actin dynamics and the effect of actin cytoskeleton modifying agents on retinoid receptor-mediated transactivation.

**Results:**

Agents that disturb the F-actin assembly or disassembly attenuated receptor-mediated transcription indicating that actin cytoskeletal homeostasis is important for retinoid receptor function. Overexpression or siRNA-induced knockdown of cofilin-1 (CFL1), a key regulator of F-actin assembly, induced the loss of receptor function. In addition, expression of either constitutively active or inactive/dominant-negative mutants of CFL1or CFL1 kinase LIMK1 induced loss of receptor function suggesting a critical role of the LIMK1-mediated CFL1 pathway in receptor-dependent transcription. Further evidence of the role of LMK1/CFL1-mediated actin dynamics, was provided by studying the effect of Nef, an actin modifying HIV-1 protein, on receptor function. Expression of Nef induced phosphorylation of CFL1 at serine 3 and LIMK1 at threonine 508, inhibited retinoid-receptor mediated reporter activity, and the expression of a number of genes that contain retinoid receptor binding sites in their promoters. The results suggest that the Nef-mediated inhibition of receptor function encompasses deregulation of actin filament dynamics by LIMK1 activation and phosphorylation of CFL1.

**Conclusion:**

We have identified a critical role of LIMK1-mediated CFL1 pathway and actin dynamics in modulating retinoid receptor mediated function and shown that LIMK1-mediated phosphocycling of CFL1 plays a crucial role in maintaining actin homeostasis and receptor activity. We suggest that T cell activation-induced repression of nuclear receptor-dependent transactivation is in part through the modification of actin dynamics.

## Background

Nuclear retinoid receptors are retinoid-induced transcription factors that mediate a wide array of cellular functions including growth, differentiation, and cell death. While the role of these receptors in immune function has been recognized early, it is only recently that retinoids have been identified to play a crucial role in T lymphocyte physiology like regulatory T cell development and suppression of inflammatory Th17 cells [1-4]. Our understanding of the mechanism of retinoid receptor function in T cells is however, not well defined. Our previous studies have provided some insights in the functioning of these receptors and have identified the relevance of epigenetic mechanisms in modulating their function during T cell signaling [5-9].

A major signaling event during T cell activation is the organization of the actin cytoskeleton and immunological synapse (IS) formation. These events are crucial for downstream signaling that culminates in effector functions and cytokine production [10,11]. Recent studies have identified CFL1, an actin binding protein, as an essential component of T cell activation that is crucial for IS formation and T cell activation. Most of the CFL1 in naive T cells is found in the inactive phosphorylated form that following TCR activation, involving accessory receptors, is dephosphorylated into an active form [10,12,13]. The active form of CFL1 binds actin and regulates F-actin dynamics. Serine/threonine kinase LIMK1 and phosphatases PP1, PP2A, slingshot 1L (SSH1L), and chronophin (CIN) are known to regulate the phosphorylated state of CFL1 [14-16].

HIV-1 gains entry into T cells by the interaction of viral proteins with receptors and co-receptors. This interaction leads to the perturbation in actin dynamics and viral replication. Recent studies have identified CFL1 as a central player in regulating the modification of the actin cytoskeleton by HIV-1 proteins gp120 and Nef [17-21]. Nef is known to interact with actin and modify actin cytoskeletal dynamics and the T cell receptor initiated signaling cascade. Nef has been also shown to inhibit IS formation and cell spreading by modifying actin function [22,23]. There is ample evidence that actin plays a functional role in nuclear transcription and disturbances in actin dynamics affect transcriptional outcome [24-32]. We have reported earlier that T cell activation silenced transcription driven by nuclear retinoid receptors and induced silencing mediator of retinoic acid and thyroid hormone receptors (SMRT)-receptor interaction (6). We also identified a dynamic balance between JNK and ERK pathways in modulating retinoid-receptor function (8). The mechanism by which T cell activation induces loss of transcription is still not completely understood. In this report we have focused our studies to investigate the role of actin cytoskeleton homeostasis and dynamics in nuclear retinoid receptor-mediated transactivation. Our results indicate a critical role of LIMK1-mediated CFL1 pathway and actin dynamics in retinoid receptor function. The data suggest that changes in the actin cytoskeletal dynamics may indeed contribute to the loss of nuclear retinoid receptor function following T cell activation.

## Results

### F-actin-modifying chemicals attenuate retinoid receptor-mediated transcription

**Figure 1:**
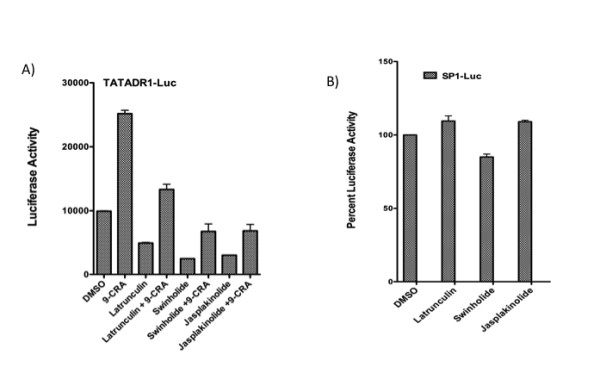
**F-actin-modifying chemicals attenuate retinoid receptor-mediated transcription**. Jurkat cells were transfected with 5.0 ug TATADR1-Luc (A) or SP1-Luc plasmids (B). After 24 h, TATADR1-Luc transfected cells were treated for 16 h with 9-*cis *retinoic acid (1.0 uM) or vehicle in the presence of DMSO, or actin inhibitors latrunculin A (50 nM), swinholide A (2.5 nM) or jasplakinolide (50 nM). After 24 h, Sp1-Luc transfected cells were treated with DMSO or actin inhibitors for 16 h. Cells were harvested and luciferase activity measured as described in Experimental Procedures.

One of the outcomes of T cell activation is remodeling of the actin cytoskeleton crucial for the formation of IS. We have previously shown that T cell activation inhibits nuclear retinoid receptor mediated function [6,8] raising the possibility that actin modification may have a role in such inhibition. To confirm the role of actin dynamics in transcriptional activation we studied the effect of actin modifying agents on receptor function. Jurkat cells were transfected with RXRE reporter plasmid and after incubation for 24 h treated for 16 h with 9-*cis *retinoic acid or vehicle in the presence of latrunculin A (50 nM), swinholide A (2.5 nM); chemicals that prevent F-actin formation, or jasplakinolide (50 nM) a compound that induces F-actin formation and stabilization. All these inhibitors were found to attenuate ligand independent and ligand-dependent transcription (Figure 1A). We used SP1-Luc reporter plasmid as a control as the transcription driven by this promoter was not significantly affected by these agents (Figure 1B). These results demonstrate that F-actin dynamics and assembly are critical for retinoid receptor-mediated transcription.

### CFL1 plays a critical role in regulating retinoid receptor function via establishing dynamic balance between F-actin stabilization and disassembly

**Figure 2:**
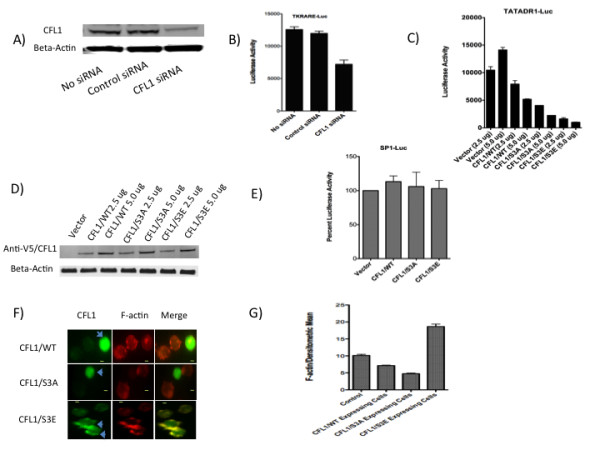
**CFL1 regulates retinoid receptor function**. A and B) Jurkat cells were transfected with 2.5 ug of TKRARE-Luc plasmid either in the presence or absence of 2.5 uM control siRNA or CFL1-specific siRNA. Cells were harvested after 36 h and lysates subject to Western blotting using antibodies to CFL1 and beta-actin (A) and luciferase activity measurement (B) as described in Experimental Procedures. C and D) Jurkat cells were transfected with 5.0 ug TATADR1-Luc in the presence of 2.5 ug and 5.0 ug of indicated plasmids. Cells were harvested after 36 h and luciferase activity measured as described in Experimental Procedures (C). Lysates were also subject o Western blotting using antibodies to anti-V5 tag (1:2,000) and beta-actin (1:10,000) (D). E) Jurkat cells were transfected with 5.0 ug of SP1-Luc plasmid in the presence of 5 ug of indicated plasmids. Cells were harvested after 36 h and luciferase activity measured as described in Experimental Procedures. SP1-Luc activity obtained in the presence of vector was normalized to 100%. F and G) Jurkat cells were transfected with CFL1/WT, CFL1/S3A, and CFL1/S3E plasmids and stained with anti-V5 Tag antibodies followed by treatment with F-actin stain as per the instructions of the manufacturer. The cells were visualized using immunofluorescent microscopy (F) and the densitometric mean intensity of F-actin from V5-CFL1 positive cells was quantified and compared to non-V5-CFL1 expressing control cells (G) using AxioVision software from Zeiss Imager D1 fluorescent microscope. Bar, 2 um.

CFL1 is known to play a central role in maintaining actin cytoskeletal dynamics by severing F-actin and allowing for re-organization and formation of new filaments [14-16]. Since our data suggested that retinoid receptor function is sensitive to changes in F-actin organization, we explored the possibility that CFL1 may play an important role in retinoid receptor activation by knocking down CFL1 protein and testing for retinoid receptor-mediated activation. Jurkat cells were transfected with reporter plasmid in the presence or absence of a control siRNA or CFL1 specific siRNA. Western blot analysis showed a nearly 75% knockdown of CFL1 protein in cells transfected with CFL1 specific siRNA as compared with cells transfected in the absence of siRNA or control siRNA (Figure 2A). When the lysates were tested for luciferase activity there was significant reduction in activity in cells transfected with CFL1-specific siRNA as compared with cells transfected in the absence of siRNA or the presence of control siRNA (Figure 2B). These results indicate that CFL1 is essential for retinoid receptor-mediated transcription. To further study the role of CFL1 in retinoid receptor activation we overexpressed CFL1 in Jurkat cells and studied its effect on transcription. The results obtained (Figure 2C and 2D) show that overexpression of CFL1/WT reduced transcriptional activation in a dose dependent manner. Taken together, knockdown and overexpression data indicate that retinoid receptor-mediated transcription is sensitive to the changes in the levels of CFL1 expression and any decrease or increase in the levels of CFL1 results in the loss of transcriptional activity.

F-actin severing activity of CFL1 is regulated by phosphorylation of the serine residue at amino acid position 3. Whereas non-phosphorylated CFL1 binds and severs F-actin, phosphorylated CFL1 is inactive and its accumulation leads to the increase in F-actin content [14-16]. Expression of the S3A mutant of CFL1 generates a protein that is not phosphorylatable and hence is constitutively active. In contrast, the S3E mutant of CFL1 mimics phosphorylated CFL1 and also functions as dominant-negative protein [16]. To identify the role of S3 phosphorylation in CFL1-regulated retinoid receptor function, Jurkat cells were transfected with reporter plasmid in the presence of CFL1/WT, CFL1/S3A, and CFL1/S3E plasmids. Surprisingly, expression of both mutants of CFL1 inhibited transcription in a dose dependent manner (Figure 2C and 2D) similar to the inhibition by CFL1/WT described above. The inhibition was more pronounced with S3A and S3E mutants as compared to CFL1/WT; the S3E mutant was most inhibitory of the three. We used SP1-Luc reporter plasmid as a control and studied the effect of CFL1/WT, CFL1/S3A, and CFL1/S3E expression on its activity. The data (Figure 2E) show that the SP1 activity was not affected by the expression of either WT or CFL1 mutants. To study the changes in the F-actin levels after transfection with CFL1/WT, CFL1/S3A, and CFL1/S3E plasmids, the cells were stained with anti-V5 Tag antibodies followed by treatment with F-actin stain. The cells were visualized (Figure 2F) and the levels of F-actin quantified (Figure 2G) using immunofluorescent microscopy. The results show that cells transfected with CFL1/WT and CFL1/S3A plasmids showed lower levels of F-actin as compared to cells not expressing the plasmid with CFL1/S3A expressing cells exhibiting lowest F-actin levels. In contrast cells transfected with CFL1/S3E plasmid showed accumulation of F-actin and expressed significantly higher levels of F-actin as compared to non-transfected cells.

Together, these results show that while CFL1 is essential for retinoid receptor function, when expressed at higher levels both active and inactive forms inhibit transcription. In other words, CFL1 regulates retinoid receptor dependent transcriptional homeostasis by maintaining a dynamic balance between F-actin stabilization and disassembly mediated by the inactive phosphorylated and the active non-phosphorylated forms of the protein.

### CFL1 kinase LIMK1 regulates retinoid receptor mediated activation

**Figure 3:**
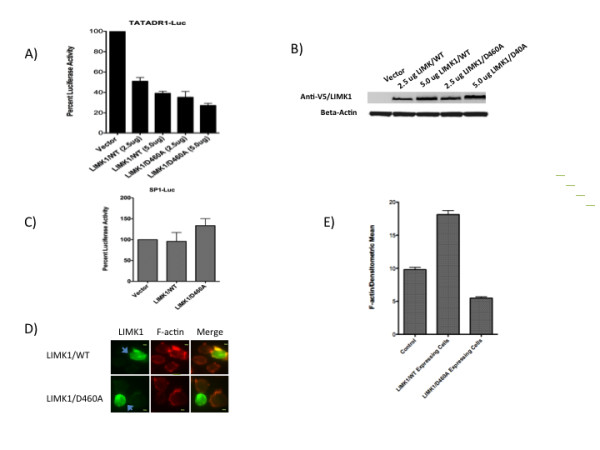
**LIMK1 regulates retinoid receptor function**. A and B) Jurkat cells were transfected with 5.0 ug TATADR1-Luc in the presence of 2.5 ug and 5.0 ug of indicated plasmids. Cells were harvested after 36 h and luciferase activity measured as described in Experimental Procedures (A). Lysates were also subject o Western blotting using antibodies to anti-V5 tag (1:2,000) and beta-actin (1:10,000) (B). C) Jurkat cells were transfected with 5.0 ug of SP1-Luc plasmid in the presence of 5 ug of indicated plasmids. Cells were harvested after 36 h and luciferase activity measured as described in Experimental Procedures. SP1-Luc activity obtained in the presence of vector was normalized to 100%. D and E) Jurkat cells were transfected with LIMK1/WT, and CFL1/D460A plasmids and stained with anti-V5 Tag antibodies followed by treatment with F-actin stain. The cells were visualized using immunofluorescent microscopy (D) and the densitometric mean intensity of F-actin from V5-LIMK1 positive cells was quantified and compared to non-V5-LIMK1 expressing control cells (E) using AxioVision software from Zeiss Imager D1 fluorescent microscope. Bar, 2 um.

LIMK1 is a threonine/serine protein kinase, a member of the LIM kinase (LIMK) family that is involved in the regulation of actin polymerization and microtubule disassembly. Although a number of proteins have been identified that are known to interact with LIMK1, CFL1 is the only known substrate for the enzyme. LIMK1 modulates actin dynamics by phosphorylating and inactivating CFL1 leading to the accumulation of F-actin[14]. The role of LIMK1 in the nuclear retinoid receptor function is unknown. Based on our observation that CFL1 plays a crucial role in regulating retinoid receptor function, we next explored the role of LIMK1 in retinoid receptor function. As shown (Figure 3A and 3B) the overexpression of LIMK1/WT in Jurkat cells inhibited retinoid receptor mediated transcription in a dose dependent manner. A LIMK1 mutant LIMK1/D460A is a kinase dead mutant that also functions as a dominant-negative inhibitor of LIMK1[33]. Transfection of Jurkat cells with reporter plasmid in the presence of LIMK1/D460A mutant plasmid also inhibited transcription driven by retinoid receptors in a dose dependent manner (Figure 3A, B). We used SP1-Luc reporter plasmid as a control and studied the effect of LIMK1/WT, and LIMK1/D460A expression on its activity. The data (Figure 3C) show that the SP1 activity was not significantly affected by the expression of the WT or mutant LIMK1. Together, these results suggest that the steady state levels of LIMK1 are important for normal retinoid receptor function. When overexpressed, LIMK1 functions as a negative regulator of retinoid receptor-mediated transcription. The inhibition of transcription exhibited by the expression of inactive and dominant negative LIMK1/D460A mutant provides further evidence for the importance of the steady state levels of the LIMK1 enzyme in regulating normal receptor function. Because overexpression and inhibition of LIMK1 will induce hyperphosphorylation and dephosphorylation of CFL1, respectively, any changes in the activity of CFL1 disturb receptor-mediated transcription. Thus LIMK1-mediated CFL1 homeostasis is crucial for receptor function. To investigate if the transfection of LIMK1/WT and LIMK1/D460A plasmids induce changes in the F-actin levels, the transfected cells were stained with anti-V5 Tag antibodies followed by staining with F-actin stain. The cells were visualized (Figure 3D) and the levels of F-actin quantified (Figure 3E) using immunofluorescent microscopy. The results show that cells transfected with LIMK1/WT plasmid showed significant accumulation and higher levels of F-actin as compared to cells not expressing the plasmid. In contrast, cells transfected with LIMK1/D460A plasmid expressed lower F-actin levels as compared to non-transfected cells. These data are consistent with the ability of LIMK1/WT expression to induce phosphorylation and inactivation of CFL1 resulting in the accumulation of F-actin, whereas expression of LIMK1/D460A has the opposite effect of increasing active CFL1 (by inhibiting phosphorylation) and inducing loss of F-actin. Together, these data indicate that LIMK1 functions as a critical regulator of retinoid receptor transcription by regulating F-actin homeostasis.

### Actin modifying HIV-1 Nef protein attenuates retinoid receptor-mediated transcription

**Figure 4:**
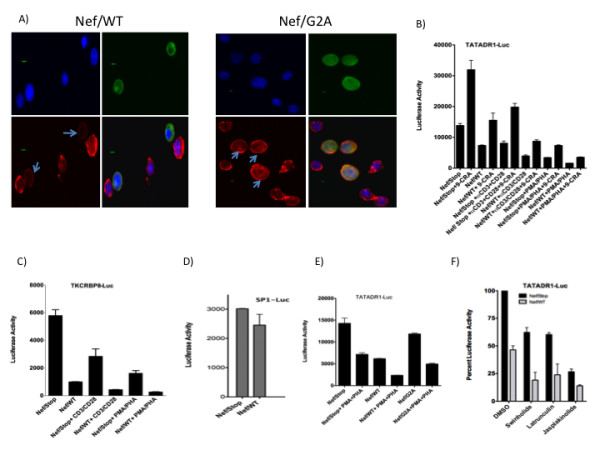
**HIV-1 Nef induces loss of F-actin assembly and inhibits retinoid receptor-mediated transcription**. A) Jurkat cells were transfected for 36 h with 5.0 ug of Nef/WT or Nef/G2A plasmids using Fugene-HD. The cells were fixed, permeabilized, and blocked with BSA as described in Experimental Procedures. Cells were incubated with anti-Nef anti-serum (1:500 in 5% BSA/PBS) for 1-2 h and labeled with FITC-conjugated secondary antibody. Cells were stained for F-actin using Alexa Fluor 555 Phalloidin and mounted in Prolong Gold Antifade Reagent with DAPI. Confocal images were obtained using an Olympus Fluoview 1000-inverted microscope. Each figure is a quadron for DAPI, Nef, F-actin, and merged images. F-actin stained Nef expressing cells are indicated by arrows. Bar, 2.5 um. B-E) Jurkat (B, E) or PBT (C) cells were transfected with 5.0 ug TATADR1-Luc or TKCRBPII-Luc plasmids respectively in the presence of 2.5 ug of indicated plasmids. After 16 h, cells were treated as indicated and luciferase activity measured 24 h later as described in Materials and Methods. D) Jurkat cells were transfected with 2.5 ug of SP1-Luc in the presence of indicated plasmids and luciferase activity measured 36 h later. F) Jurkat cells were transfected with 5.0 ug TATADR1-Luc in the presence of 2.5 ug of indicated plasmids. After 16 h, cells were treated with indicated compounds and luciferase activity measured 24 h later as described in Experimental Procedures.

Recent studies have identified CFL1 as a central player in regulating the modification of the actin cytoskeleton by HIV-1 proteins gp120 and Nef [17-21]. Nef is known to interact with actin and modify actin cytoskeletal dynamics and the T cell receptor initiated signaling cascade. Nef also has been shown to inhibit IS formation and cell spreading by modifying actin function [22,23]. Nef functions by binding to the cell membrane and incorporation into lipid rafts through myristoylation. We first studied the effect of wild-type (WT) and myristoylation (G2A) mutant of HIV-1 Nef on F-actin assembly using immunofluorescence microscopy. Jurkat cells were transfected with Nef/WT and Nef/G2A plasmids and 36 h later stained with anti-Nef antibody and F-actin specific fluorophore-labeled phalloidin. As shown in Figure 4A expression of WT but not G2A mutant of Nef induced loss of characteristic F-actin ring assembly in these cells. Next, we investigated the effect of Nef on retinoid receptor mediated transcription. Jurkat and PBT cells transfected with either RXRE or RARE containing luciferase-based reporter plasmids in the presence of Nef-expressing plasmid showed a significant loss of reporter activity when compared to cells transfected with a non-expressing Nef control plasmid (Figure 4B and Figure 4C).

SP1-Luc reporter was used a control as Nef did not have significant effect on the activity of this promoter (Figure 4D). To study if the T cell activation signals synergized with Nef in inhibiting transcription, cells were transfected with Nef expressing plasmid in the presence of antibodies to T cell receptors CD3 and CD28 or treatment with PMA+PHA (Figure 4B and Figure 4C). The data show that activation significantly enhanced Nef induced loss of receptor activity. In order to identify the role of myristoylation, G2A mutant of Nef was then tested in reporter assays. The results show (Figure 4E) that loss of myristoylation significantly reduced the ability of Nef to induce inhibition. Next, we studied the effect of F-actin-modifying chemicals on transcriptional activation in the presence of Nef to see if the latter cooperated with the chemicals in inhibiting transcription. Jurkat cells were transfected with reporter plasmid either in the presence of Nef/WT or Nef/Stop plasmids for 24 h followed by treatment with chemicals. The results (Figure 4F) demonstrate that Nef is significantly more inhibitory in the presence of actin-modifying chemicals. Nef cooperating with actin-modifying chemicals points to the modulation of F-actin assembly as a possible mechanism for Nef-induced inhibition of transcriptional activation.

### Nef inhibits the expression of cellular genes containing retinoid receptor binding sites in their promoters

Nef was also found to inhibit RXRE or RARE containing luciferase-based reporter activity in HEK-293 cells (data not shown) indicating that Nef inhibition of retinoid receptor function is not limited to T cells alone. To confirm the data obtained by the reporter assays and identify retinoid receptor-dependent cellular genes that are inhibited by Nef expression, we transfected 293 cells with Nef/Stop and Nef/WT plasmids and the effect on cellular transcriptome was analyzed by gene array. The data identified that Nef inhibited the expression of five genes that contain retinoid receptor binding sites in their promoters (Table 1). IFIT1, CEBPB, and CRABP2 are known retinoic acid inducible genes [34-36]whereas RBM8A and ACOT2 were found to contain DR2 (RARE) and DR1 (RXRE) consensus sequence elements, respectively, within their promoter regions upstream of the transcription start site. Real-Time RT-PCR was used to confirm the inhibition of gene expression.

**Table 1:** Nef-induced inhibition of retinoid receptor-mediated gene expression.

Name	Accession #	RXRE/RARE site in the promoter	Fold Change		Reference
			Gene Array	Real-Time-RT-PCR	
IFIT1	NM_001548	Retinoic acid-inducibility known	-2.12	-8.30	[34]
RBM8A	BC071577	AGGTCAGGAGTTCA	-1.82	-1.61	-
ACOT2	NM_006821	AGCTCAGAGGTCA	-1.75	-1.32	-
CEBPB	NM_005194	Retinoic acid-inducibility known	-1.50	-1.60	[35]
CRABP2	NM_001878	Retinoic acid-inducibility known	-1.32	-1.74	[36]

293 cells were transfected with Nef/Stop and Nef/WT plasmids for 36 h. Total RNA was isolated and processed for gene array as described in Experimental Procedures. Five genes whose expression was significantly inhibited by Nef were identified to be either known retinoic acid-inducible genes or contained RAR/RXR binding sites in their promoters. IFIT1, CEBPB, and CRABP2 were found to be known retinoic acid-inducible genes whereas. RBM8A and ACOT2 were found to contain putative RARE (DR2) and RXRE (DR1) sites respectively in their promoter regions upstream of the transcription start site. The inhibition of gene expression was validated by Real-Time RT-PCR.

### Nef inhibition of retinoid receptor function is mediated by LIMK1-dependent CFL1 pathway

**Figure 5:**
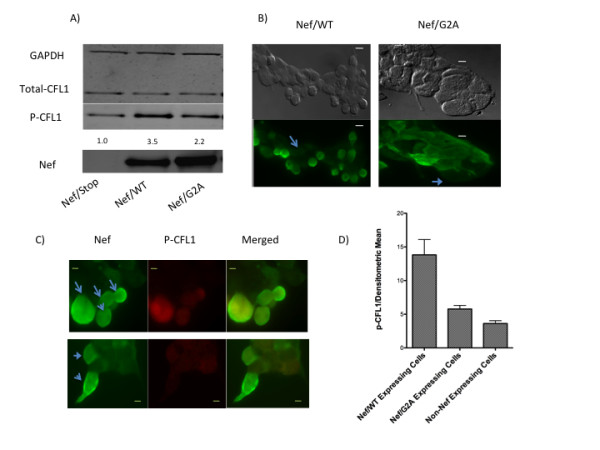
**Nef induces CFL1 phosphorylation**. A) 293 cells were transfected with Nef/Stop, Nef/WT or Nef/G2A plasmids for 36 h. Cell lysates were then subject to Western blotting using antibodies to Nef (1:2,000), total CFL1 (1:1,000), phospho-serine-3-CFL1 (1:2,000), and GAPDH (1:5,000). The intensity of the phospho-serine-3-CFL1 bands was quantified and normalized to Nef/Stop that was assigned a value of 1.0. B and C) 293 cells transfected with either Flag-tagged Nef/WT or Flag-tagged Nef/G2A plasmids were fixed, permeabilized, and blocked with BSA (as described in Experimental Procedures), incubated with anti-Flag (B) or anti-Flag and anti-phospho-serine-3-CFL1 antibodies (C) (1:500 in 5% BSA/PBS) for 2 h, and stained with FITC-conjugated (B) or FITC-conjugated and Alex-Fluor-555 labeled secondary antibodies (C). The cells were mounted in Prolong Gold Antifade Reagent before immunofluorescence microscopy. Fig 5B also shows the phase contrast image of 293 cells expressing Flag-tagged Nef/WT exhibiting "rounding off" phenotype unlike cells expressing Flag-tagged Nef/G2A. The arrows in Fig. 5B identify non-Nef expressing cells. The panels in Fig 5C are for Nef, p-CFL1, and merged images. The arrows in Fig. 5C identify Nef expressing cells. Bar, 5 um. D) The densitometric mean intensity of p-CFL1 stained cells was quantified from Nef/WT and Nef/G2A transfected cells and compared to non-Nef expressing cells.

LIMK1 functions with CFL1 to control the assembly of actin. To understand the mechanism of Nef-induced modulation of actin-dynamics and nuclear receptor-mediated function, we next investigated the possibility that overexpression of Nef could regulate CFL1 activity by modifying the level of CFL1 phosphorylation. These studies were performed in 293 cells that also exhibited F-actin changes after transfection with Nef (data not shown). Cells were transfected with plasmids expressing Nef/WT or Nef/G2A mutant. Western blot analysis of the extracts collected after 36 hr of transfection showed that cells transfected with Nef/WT expressed significantly higher levels of phosphorylated CFL1 as compared to cells transfected with control or Nef/G2A plasmids (Figure 5A). The levels of total CFL1 remained unchanged. These data show that Nef induces CFL1 phosphorylation and myristoylation of Nef protein is crucial for this activity of Nef.

We next explored the effect of Nef on CFL1 phosphorylation using immunofluorescence microscopy. 293 cells were transfected with either Flag-tagged Nef/WT or Flag-tagged Nef/G2A plasmids. After 36 h cells were stained with anti-Flag and anti-phospho CFL1 antibodies. Microscopic study revealed that Nef expression induced morphological changes in 293 cells. Although still attached to the surface, Nef-expressing cells appeared more rounded in shape than cells not expressing Nef or cells that expressed the G2A mutant of Nef (Figure 5B). In addition, Nef-expressing and rounded cells showed significantly higher levels of phospho-CFL1 staining as compared to cells not expressing Nef and cells expressing the G2A mutant protein (Figure 5C and 5D). These results confirm Western blotting results that expression of the myristoylated form of Nef induces CFL1 phosphorylation.

**Figure 6:**
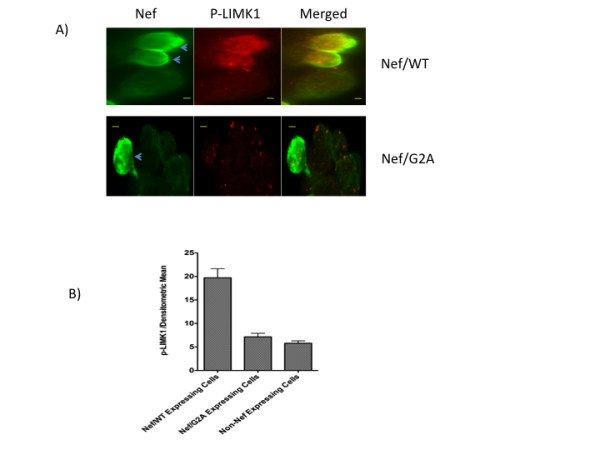
**Nef induces LIMK1 activation**. 293 cells were transfected with either Flag-tagged Nef/WT (A) or Flag-tagged Nef/G2A (B) plasmids. After 36 h cells were fixed, permeabilized, and blocked with BSA as described in Experimental Procedures. Cells were incubated with anti-Flag tag (1:500 in 5% BSA/PBS) and phospho-specific LIMK1 (1:250 in 5% BSA/PBS) antibodies for 2 h and labeled with FITC-conjugated (Flag) and Alex-Fluor-555 labeled (phospho-CFL1) secondary antibodies. The cells were mounted in Prolong Gold Antifade Reagent before immunofluorescence microscopy. The panels in the figure are for Nef, p-LIMK1, and merged images. Arrows identify Nef expressing cells. Bar, 5 um. B) The densitometric mean intensity of p-LIMK1 stained cells was quantified from Nef/WT and Nef/G2A transfected cells and compared to non-Nef expressing cells.

Based on our findings in this study that overexpression of the CFL1 kinase LIMK1 inhibits retinoid receptor-mediated transactivation and Nef induces phosphorylation of CFL1, it was logical to hypothesize that Nef attenuates retinoid receptor mediated activation by inducing the activation of LIMK1 which in turn increases CFL1 phosphorylation. To test the hypothesis that Nef induces activation of LIMK1, we transfected 293 cells with either Flag-tagged Nef/WT or Flag-tagged Nef/G2A plasmids for 36 h and studied LIMK1 phosphorylation at Thr508. Phosphorylation at Thr508 is known to activate the LIMK1 enzyme [33]. Immunofluorescence analysis of the transfected cells using antibodies specific to phospho-LIMK1 (antibodies to phospho-peptide corresponding to residues surrounding Thr508 of LIMK1) revealed that Nef expressing cells, that also showed the "rounding off" phenotype, exhibited significantly higher levels of phospho-LIMK1 staining as compared to cells not expressing Nef (Figure 6). In contrast, cells expressing the G2A mutant protein that did not show the "rounding off" phenotype, exhibited phospho-LIMK1 levels similar to the cells not expressing the Nef protein. These results confirm the hypothesis that Nef induces the activation (phosphorylation) of LIMK1. In addition, the data also reveal that myristoylation of Nef is necessary to induce the "rounding off" phenotype and activation of LIMK.

## Discussion

The actin cytoskeleton undergoes major changes during T lymphocyte migration, antigen-driven activation, apoptosis, and infection with retroviruses [10-14,17,19-21]. These events result in the activation of various signaling components that are assembled and brought into the close proximity by the actin cytoskeleton. Actin cytoskeletal homeostasis also regulates gene expression by modulating chromatin remodeling, mRNA processing, and nuclear export [24-30,32]. Thus actin pathways regulate cellular functions by the dual processes of modulating signaling and also directly participating in nuclear transcription. It is known that retinoid receptor function is attenuated during T cell activation [6,8], a phenomenon that involves actin remodeling, suggesting that actin modification may play a role in such inhibition. In this study we set out to investigate the role of actin cytoskeleton homeostasis and dynamics in retinoid receptor-mediated transcription. Latrunculin A, swinholide A, compounds that prevent F-actin formation, or jasplakinolide, a chemical that induces F-actin formation and stabilization, were all found to attenuate transcription to various levels. These results point out the importance of actin homeostasis in retinoid receptor-mediated activation and indicate that any disturbances in actin dynamics and the F-actin homeostasis attenuate retinoid receptor-dependent transcription. Electrophoretic mobility shift assay did not reveal any significant loss of RARE or RXRE probe binding activity in response to F-actin modifying agent (data not shown) suggesting that mechanisms other than the effect on DNA binding are involved in transcriptional inhibition induced by F-actin disruption.

CFL1 is a key F-actin remodeling protein that severs F-actin and thereby allows addition and assembly of new actin filaments, the so-called actin-treadmilling [15]. Both siRNA-mediated knockdown and overexpression of CFL1 attenuated retinoid receptor function showing that CFL1 homeostasis plays a critical role in receptor activity. This was further evident from the data obtained with constitutively active (S3A) and inactive/dominant-negative (S3E) mutants of CFL1. Whereas the transfection of cells with CFL1/WT and S3A mutant induced loss of F-actin content in the cells expressing these proteins, transfection with S3E mutant increased F-actin content consistent with the ability of WT and S3A mutant to severe F-actin and S3E mutant to increase F-actin content. Loss of transcriptional activation by the expression of S3A and S3E mutants of CFL1, in addition to CFL1/WT, further emphasizes the importance of actin homeostasis in the regulation of retinoid receptor-mediated transcription.

Interestingly, CFL1 overexpression has also been shown to negatively regulate glucocorticoid receptor function [31] and thrombin-induced NF-kappaB activity [37] suggesting that CFL1 homeostasis is also essential for the activities of steroid hormone-mediated nuclear receptors as well as other transcription factors besides retinoid receptors reported in this study.

The role of LIMK1 in the regulation of nuclear hormone receptor-dependent activation is unknown. Here we have shown that when overexpressed LIMK1 functions as a negative regulator of retinoid receptor function. The expression of an enzymatically inactive D460A mutant of LIMK1, that also functions as a dominant-negative protein, also inhibited receptor activity. These data suggest that physiological levels of LIMK1 are crucial for receptor function due to its ability to regulate CFL1 and actin cytoskeletal homeostasis. Overexpression of LIMK1 and loss of LIMK1 activity (D460A mutant expression) increased and decreased F-actin content respectively, and deregulated actin dynamics and homeostasis so crucial for retinoid receptor activity.

A number of previous studies have demonstrated that Nef expression disturbs F-actin cytoskeleton [17-23]. We confirmed these observations in this study and found that Nef expression induced loss of the characteristic actin ring structure. In addition, we report that Nef inhibited retinoid receptor mediated transcription. The gene array analysis of Nef-transfected 293 cells identified inhibition of endogenous expression of a number of genes that contain retinoid receptor binding sites in their promoters. IFIT1, CEBPB, and CRABP2 are known retinoic acid inducible genes [34-36] whereas RBM8A and ACOT2 were found to contain DR2 (RARE) and DR1 (RXRE) consensus sequence elements, respectively, within their promoter regions upstream of the transcription start site.

The role of myristoylation in reducing the receptor function correlated well with the inability of this Nef mutant to induce loss of F-actin ring structure. The conclusion that the Nef-mediated loss of transcriptional activation is a consequence of disturbance in the actin dynamics was supported by the observation that the presence of Nef exhibited a cooperative effect on the loss of transcription induced by the actin-modifying agents. These data show that retinoid receptor mediated transcription is tightly controlled by actin cytoskeletal homeostasis and any disturbances in F-actin dynamics that may include changes in F-actin architecture, length, and content affect receptor-driven transcription.

In order to gain further insight to understand the role of actin pathways in modulating retinoid receptor-mediated function, we next studied the mechanism by which Nef inhibits actin dynamics. We have shown that cells expressing Nef had higher levels of phospho-CFL1 suggesting a link between Nef mediated phosphorylation of CFL1 and inhibition of retinoid receptor-mediated activation. By inhibiting CFL1 activity Nef protein disturbs F-actin homeostasis that is crucial for normal retinoid receptor function. Nef induced phosphorylation of CFL1 has also recently been reported by others [20,38] indicating that modification of CFL1 activity is an important mechanism by which Nef modulates actin-cytoskeletal dynamics and cellular functions. The mechanism underlying the phosphorylation of CFL1 by Nef is not known. Although phospho-CFL1 appears to colocalize with Nef (Figure 5), but Nef did not interact with CFL1 in immunoprecipitation experiments (unpublished observation). Since Nef lacks any kinase activity, phosphorylation of CFL1 by Nef is probably induced due its activation and association with other kinases and/or adapter proteins like VAV. Nef has been reported to associate with p-21 associated kinases (PAK) 1 and 2 and induce activation of these serine/threonine kinases [39-41]. PAKs are effectors of RAC and CDC42 GTPases that are known for their role in actin dynamics. PAK1 is an upstream kinase for LIMK1 that binds and activates LIMK1 by phosphorylating Thr508 within the activation loop, resulting in a marked increase in its activity to phosphorylate CFL1 [42]. In fact treatment of Jurkat cells with PAK inhibitor IPA3 was able to induce retinoid receptor transcription and reduce p-CFL1 levels (data not shown) indicating that PAK-mediated CFL1 phosphocycling plays an important role in retinoid receptor function.

The ability of Nef to inhibit transcription and induce CFL1 phosphorylation suggested that Nef might function by activating LIMK1 thereby inducing CFL1 phosphorylation. Data presented here support this hypothesis and demonstrated that the expression of myristoylated Nef induced significant phosphorylation of LIMK1 at Thr508. How Nef induces phosphorylation of LIMK1 is not known but Nef-mediated activation of PAK1 that is known to directly phosphorylate LIMK1 [42] remains a possibility. We also found that expression of Nef induces the "rounding off" phenotype in cells that is indicative of cell cycle changes seen in mitotic cells [43,44]. This rounding phenotype induced by Nef was associated with increased phospho-CFL1 and phospho-LIMK1 levels (Figures 5 and 6). Although a number of previous studies have shown that there is a transient net change in the phospho-CFL1 levels and increase in the phospho-LIMK1 levels during mitosis and cytokinesis [45-48], but our cell cycle studies did not reveal any significant changes in the number of cells in mitotic phase in cells transfected with Nef (data not shown). It is possible that the "rounding off" phenotype seen in this study may reflect the morphological changes in cells due to disturbance in F-actin dynamics and not the presence of mitotic cells.

Our data strongly indicates that Nef is a modulator of LIMK1 and CFL1 phosphorylation that modifies the function of these two proteins and inhibits retinoid receptor transcription by interfering in actin architecture. This is further emphasized in our overexpression data using various CFL1 and LIMK1 mutants that induce loss of transcription and disturbance in the actin dynamics. Data obtained with Nef protein not only confirm the role of actin dynamics in retinoid receptor transcription, but also provide mechanistic information. Although we cannot rule out the possibility that HIV-Nef inhibits receptor function through additional mechanisms besides modifying CFL1 phosphocycling, modification of actin dynamics by Nef and the crucial role of actin homeostasis in receptor function provide evidence that Nef inhibits receptor function largely by inhibiting actin dynamics.

In conclusion, we have described a critical role of actin-cytoskeleton dynamics in normal retinoid receptor-mediated function and found that LIMK1-mediated phosphocycling of CFL1 plays a crucial role in maintaining actin homeostasis and receptor activity. Our studies however, do not rule out the importance of LIMK2 in retinoid receptor function. We have identified HIV-1 Nef protein as an inhibitor of receptor function by virtue of its LIMK1-meditaed CFL1 modifying activity and used it as tool to study the importance of actin dynamics in receptor function. Further studies are needed to identify the mechanism by which disturbances in actin dynamics directly alter the ability of retinoid receptors to activate transcription in the nuclear compartment. A number of studies have demonstrated that nuclear actin exists in dynamic equilibrium between monomeric and polymeric forms, indicating that polymerization of monomeric G-actin is an essential feature of actin dynamics in the nucleoplasm [49,50]. A recent report has shown that most of the G-actin pool in the nucleus is bound to CFL suggesting the importance of CFL in the regulation of nuclear function [51]. LIMK1 [45-48] and CIN are known to have nuclear functions [47,52].

CFL1 is an essential component of T cell activation process and is crucial for IS formation. Most of the CFL1 in naive T cells is found in the inactive phosphorylated form that following TCR activation, is dephosphorylated into an active form [10,12,13]. Our previous studies have revealed that retinoid receptor function is inhibited during T cell activation [6,8] suggesting that actin modification may play a role in such inhibition. Data presented here strongly suggests that changes in the actin cytoskeletal dynamics may indeed contribute to the loss of nuclear retinoid receptor function following T cell activation. Future studies should reveal the relationship between CFL1-mediated IS formation and modulation of retinoid receptor function.

## Conclusions

We have described a critical role of actin-cytoskeleton dynamics in retinoid receptor-mediated function and shown that LIMK1-mediated phosphocycling of CFL1 plays a crucial role in maintaining actin homeostasis and receptor activity. We have identified HIV-1 Nef protein as an inhibitor of receptor function by virtue of its LIMK1-meditaed CFL1 modifying activity and used it as tool to study the importance of actin dynamics in receptor function. Our previous studies have revealed that retinoid receptor function is inhibited during T cell activation suggesting that actin modification may play a role in such inhibition. Data presented here strongly suggests that changes in the actin cytoskeletal dynamics may indeed contribute to the loss of nuclear retinoid receptor function following T cell activation.

## Methods

### Cells and reagents

T lymphocyte leukemia Jurkat cell line (clone E6-1) was maintained in RPMI 1640 medium (Biowhittaker, Frederick, MD) supplemented with 10 mM HEPES buffer, 2 mM L-glutamine, and 1% Penn/Strep, and 10% fetal bovine serum (Hyclone, Logan, UT). Human peripheral blood mononuclear cells, obtained by lymphapheresis of healthy donors, were purified by Ficoll density gradient centrifugation. Purified peripheral blood mononuclear cells were treated with phytohemagglutinin and interleukin-2 for 2 days in AIM-V medium (Invitrogen, Carlsbad, CA) supplemented with 10% fetal bovine serum. The cells were washed to remove phytohemagglutinin and maintained in interleukin-2 as described earlier [6]. In this report, these cells will be referred to as proliferating peripheral blood T (PBT) cells. PBT cells were 98% CD3-positive as monitored by flow cytometry. HEK-293 cells were maintained in DMEM medium containing 10% fetal bovine serum. Latrunculin A, swinholide, and jasplakinolide were from EMD Biosciences (Gibbstown, NJ). HIV-Nef antibody was obtained from the NIH AIDS Research and Reference Reagent Program. Antibodies to CFL1 and phospho-threonine-508 LIMK1 were purchased from Santa Cruz Biotechnology, Inc (Santa Cruz, CA). Antibodies to beta-actin and GAPDH were from Abcam (Cambridge, MA). Alexa Fluor 555 Phalloidin, Prolong Gold Antifade Reagent with DAPI, and antibodies to phospho-serine-3 CFL1 were obtained from Invitrogen.

### Western blot

Protein extracts were electrophoresed in a 10-12% NuPAGE Bis Tris Gel using NuPAGE MES-SDS running buffer (Invitrogen, Carlsbad, CA), and transferred to a PVDF membrane using XCell Blot Module (Invitrogen). Protein was detected using fluorophore labeled secondary antibodies (1:7,500) and the Odyssey Infrared Imaging System (Li-cor Biotechnology, Lincoln, Nebraska).

### Plasmids, transfections, and RNA interference (RNAi)

Retinoid receptor mediated activation was studied by transient transfection using RXRE containing luciferase plasmids, CRBPII-TK-Luc or TATA-DR1-Luc and RARE containing plasmid TKRARE-Luc (also referred to as reporter plasmids in this report) as described [6,8]. CRBPII-TK-Luc or TATA-DR1-Luc plasmids are specific for RXR:RXR homodimers and RXR:RAR heterodimers whereas TKRARE-Luc reporter is specific for RXR:RAR heterodimers. Sp1-Luc reporter vector was from Affymetrix, Inc. (Santa Clara, CA). Human CFL1 and LIMK-1 cDNA clones were obtained from the Dana-Farber/Harvard Cancer Center DNA Resource Core (Boston, MA). The cDNAs from these plasmids were cloned into pLenti7.3/V5-DEST Gateway Vector (Invitrogen). CFL1 mutants S3A and S3E and LIMK-1 mutant D460A were created by mutagenesis. All mutations were verified by DNA sequencing and expression confirmed by Western blot analysis. HIV-1 Nef expressing plasmid p96ZM651nef-opt was obtained from the NIH AIDS Research and Reference Reagent Program. Myristoylation (G2A) mutant and a non-expressing control plasmid (Nef/Stop, containing two stop codons at amino acid positions 5 and 13) were created using mutagenesis and verified by sequencing. Flag-tagged Nef/WT and Flag-tagged Nef/G2A plasmids were constructed using PCR by introducing 3× Flag sequence at the C-terminal end of the Nef sequence from Nef/WT and Nef/G2A plasmids. CFL1 expression was knocked down in Jurkat cells by short interfering RNA (siRNA) purchased from Applied Biosystems. Jurkat cells were transfected by electroporation using a Gene Pulser II (Bio-Rad, CA) at 0.250 kV and 975 uF as described [7]. PBT cells were electroporated using a T cell Nucleofection kit from Amaxa Biosystems (Cologne, Germany) according to the manufacturer's instructions as described [5]. In some experiments cells were treated with antibodies to CD3 (5.0 ug/ml), antibodies to CD28 (1.0 ug/ml), PMA (50 ng/ml), PHA (2.5 ug/ml), and 9-*cis *retinoic acid (9-CRA) (1.0 uM) following 16 h of transfection. Twenty hours after treatment cells were harvested for luciferase measurement. Luciferase activity was normalized to protein concentrations in the extracts. The values in the figures represent the mean of three independent experiments with standard error calculated for each value.

### Fluorescence microscopy

Jurkat cells were transfected with plasmids using the Fugene HD transfection reagent from Roche (Indianapolis, IN). After 36 h cells were washed with PBS and incubated on poly-L-Lysine coated chamber slides for 1 hr. 293 cells were grown in chamber slides, transfected with plasmids using Fugene-HD, and incubated for 36 h. Cells were washed with PBS and fixed with 4% formaldehyde for 30 min and permeabilized with 0.1% Triton X-100 for 10 min. Cells were blocked with 5% BSA/PBS, stained with primary antibodies for 1-2 h, washed with PBS and labeled with dye-conjugated secondary antibodies (1:2,000). F-actin staining was performed using Alexa Fluor 555 Phalloidin. Cells were mounted in Prolong Gold Antifade Reagent with DAPI (Invitrogen). Immunofluorescence and laser-based confocal images were obtained using an Axioimager D1 Zeiss microscope and an Olympus Fluoview 1000 inverted microscope respectively. The densitometric mean intensity of F-actin stain was quantified using AxioVision software from Zeiss Imager D1 fluorescent microscope.

### Gene Array and Real-time RT-PCR

293 cells were transfected in three different experiments (each time in duplicate) with Nef/Stop and Nef/WT plasmids for 36 h using Fugene-HD. Total RNA was isolated using Qiagen (Germantown, MD) RNAeasy kit by following manufacture's protocol. 250 ng total RNA was used for the Affymetrix whole transcript sense target labeling assay. The terminal labeled products were fragmented and analyzed on Affymetrix (Santa Clara, CA) human Exon 1.0 ST arrays. The data were analyzed with Partek (St. Louis, Missouri) using the GC content and probe sequence adjusted RMA normalization. Genes were summarized by Turkey's biweight method. The significant genes were selected by P value less than 0.05 and fold change 1.3. The array data was submitted to GEO repository under accession number GSE31665. For Real-Time RT-PCR total RNA was reverse transcribed with Superscript II reverse transcriptase (Invitrogen) in the presence of 2.5 μM random hexamers. The products of reverse transcription were subject to Real-Time PCR with gene-specific primers using 7500 Fast Real-Time PCR System and Fast SYBR Green Master Mix from Applied Biosystems. The comparative threshold cycle method was used to calculate the relative gene expression.

### Statistical analysis

The results obtained from three independent experiments were expressed as mean ± SD. Statistical analysis was assessed by Student's *t *test. A value of *p *< 0.05 was considered significant.

## Authors' contributions

MI and VN conceived the study. MI wrote the manuscript and carried out IF, and transcription studies. VN evaluated the data and revised the manuscript. LB carried out reporter assays with Nef constructs. MB carried out the gene array experiments. XZ, JY, DH, and RA participated in coordination and bioinformatic analysis of the gene array studies. All authors read and approved the final manuscript.
