# Correction: LIM kinase 1 - dependent cofilin 1 pathway and actin dynamics mediate nuclear retinoid receptor function in T lymphocytes

**DOI:** 10.1186/1471-2199-13-14

**Published:** 2012-04-12

**Authors:** Mohammad Ishaq, Bor-Ruei Lin, Marjorie Bosche, Xin Zheng, Jun Yang, Dawei Huang, Richard A Lempicki, Angelica Aguilera-Gutierrez, Ven Natarajan

**Affiliations:** 1 Laboratory of Molecular Cell Biology, SAIC-Frederick, National Cancer Institute, Frederick, MD 21702, USA; 2 Laboratory of Immunopathogenesis and Bioinformatics, SAIC-Frederick, National Cancer Institute, Frederick, MD 21702, USA

## Correction

Initially Dr Angelica Aguilera-Gutierrez was not included in the authors list of an article published in BMC Molecular Biology (2011, **12:**41) by Ishaq et al [1]. All the authors agree that Dr Aguilera-Gutierrez meets the criteria for authorship, and should be considered an author of the article [1].

## Authors' contributions

MI and VN conceived the study. MI wrote the manuscript and carried out IF, and transcription studies. VN evaluated the data and revised the manuscript. LB carried out reporter assays with Nef constructs. MB carried out the gene array experiments. AA-G carried out the reporter assays with Nef constructs and Western blots. XZ, JY, DH, and RA participated in coordination and bioinformatic analysis of the gene array studies. All authors read and approved the final manuscript.

## References

[B1] Ishaq M Lin BR Bosche M Zheng X Yang J Huang D Lempicki RA Natarajan V LIM kinase 1 - dependent cofilin 1 pathway and actin dynamics mediate nuclear retinoid receptor function in T lymphocytes BMC Mol Biol 2011 12 41 10.1186/1471-2199-12-41 21923909 PMC3187726

